# Associations Between Hip Mobility and Pain in Chronic Low Back Pain Using IMU and Markerless Motion Capture

**DOI:** 10.3390/s26123713

**Published:** 2026-06-11

**Authors:** Elpida Foti, Athanasios Triantafyllou, Nefeli Maria Tsirmpini, Panagiotis Koulouvaris, Charilaos Tsolakis, Apostolos Z. Skouras, Eleni-Maria Kaframani, Konstantina Karnarou, Sofia A. Xergia, Sofia Lampropoulou, Panagiota Papadea, Nikolaos Tachos, Georgia S. Karanasiou, Maria Kyriakidou, Sophia Stasi, Panagiotis Gkrilias, Georgios Papagiannis

**Affiliations:** 1 Sports Excellence, 1st Department of Orthopaedic Surgery, School of Medicine, National and Kapodistrian University of Athens, 12462 Athens, Greece; athanat@gmail.com (A.T.); 02nefelie@gmail.com (N.M.T.); info@drkoulouvaris.gr (P.K.); apostolis.sk@gmail.com (A.Z.S.); marialenak34@gmail.com (E.-M.K.); karnaroukonna@gmail.com (K.K.); grpapagiannis@yahoo.gr (G.P.); 2 Biomechanics Laboratory, Department of Physiotherapy, University of the Peloponnese, 23100 Sparta, Greece; m.kyriakidou@uop.gr (M.K.); stasis@go.uop.gr (S.S.); p.gkrilias@uop.gr (P.G.); 3 Sports Performance Laboratory, School of Physical Education & Sports Science, National and Kapodistrian University of Athens, 17237 Athens, Greece; 4 Department of Physiotherapy, School of Health Rehabilitation Sciences, University of Patras, 26504 Patra, Greece; sxergia@upatras.gr (S.A.X.); lampropoulou@upatras.gr (S.L.); 5 Department of Physiotherapy, School of Health Rehabilitation Sciences, University of Peloponnese, 23100 Sparta, Greece; peggy.papadea@gmail.com; 6 Unit of Medical Technology and Intelligent Information Systems, University of Ioannina, 45110 Ioannina, Greece; ntachos@gmail.com (N.T.); g.karanasiou@gmail.com (G.S.K.)

**Keywords:** inertial measurement units (IMUs), markerless motion capture, human motion analysis, chronic low back pain, sensor-based movement assessment

## Abstract

**Highlights:**

**What are the main findings?**

- Hip flexion measured at the point of lumbar pain provocation provides clinically meaningful information in individuals with chronic nonspecific low back pain (CNLBP).
- Improvements in hip flexion were moderately associated with reductions in low back pain intensity but were not associated with changes in functional disability measured by the Oswestry Disability Index (ODI).
- IMU-based systems demonstrated higher measurement precision, whereas markerless motion capture systems reliably detected movement trends.

**What is the implication of the main findings?**

- The findings support the biomechanical relevance of hip mobility in lumbopelvic mechanics and its contribution to pain modulation in CNLBP.
- Symptom-specific assessment of movement may provide more clinically relevant information than conventional range-of-motion measurements alone.
- Combining objective motion analysis with symptom-specific evaluation may facilitate more individualized rehabilitation planning in musculoskeletal care, while markerless systems may offer a scalable and accessible alternative for clinical implementation.

**Abstract:**

**Introduction**: Chronic non-specific low back pain (CNLBP) is associated with altered lumbopelvic mechanics and impaired hip mobility. This study examined whether changes in pain-provoking hip flexion are associated with changes in low back pain and assessed agreement between inertial measurement units (IMUs) and a markerless motion capture system. **Methods**: Thirty-six patients with CNLBP completed a longitudinal repeated-measures rehabilitation protocol consisting of approximately 13 physiotherapy sessions over a period of up to 6 weeks. Active hip flexion was assessed in the symptomatic limb (the limb provoking lumbar pain). Hip flexion was recorded during the same movement trial using IMUs and a markerless system. Pain and disability were assessed using the Visual Analogue Scale and Oswestry Disability Index. **Results**: Improvements in hip flexion were moderately associated with pain reduction (markerless: r = −0.52; IMU: r = −0.57), with negligible associations with disability. Markerless and IMU measurements showed a strong correlation (r = 0.87), while Bland–Altman analysis showed consistent underestimation by the markerless system (bias = −3.67°). **Conclusions**: Symptom-specific hip mobility is associated with pain reduction in CNLBP, highlighting the role of lumbopelvic biomechanics. IMUs demonstrated higher consistency, while markerless systems offered a more accessible alternative for clinically meaningful movement assessment.

## 1. Introduction

Chronic non-specific low back pain (CNLBP) has become a significant burden on both healthcare systems and global productivity, making it one of the most common causes of disability worldwide [1]. According to estimates, up to 70% of adults in developed countries will experience low back pain at some point in their lives [2]. This trajectory frequently results in long-term functional impairment and a reduced ability to work [3]. To understand the management of CNLBP, it is necessary to recognize its multifactorial nature, in which the clinical presentation is influenced by the complex interplay of biomechanical, psychological, and social factors [4].

Increasing evidence indicates that altered lumbopelvic kinematics play a role in individuals with CNLBP. Limited hip range-of-motion, particularly during active hip flexion, has been linked to compensatory lumbar spine movements and increased mechanical strain [5]. Clinical evidence supports this link, demonstrating that individuals with limited hip mobility often have increased pain intensity and impaired functional capacity compared to those without hip involvement [6,7]. Moreover, diminished lumbar spine mobility and compromised coordination between the hip and lumbar sections have been associated with the persistence of pain and disability [8].

Patient-reported outcome measures such as the Oswestry Disability Index (ODI) and the Visual Analogue Scale (VAS) are widely used to assess functional limitations and pain intensity, respectively. The ODI provides a validated measure of disability related to daily activities [9], while the VAS offers a reliable quantification of perceived pain [10]. However, the relationship between objective biomechanical changes and subjective clinical outcomes remains insufficiently explored.

In recent years, wearable Inertial Measurement Units (IMUs) have been widely adopted as reliable tools for motion analysis, providing accurate measurement of joint kinematics in clinical and real-world settings [11,12,13]. Meanwhile, advances in computer vision and machine learning have enabled markerless motion capture systems that assess movement without requiring wearable sensors [4,14,15,16]. Despite the evident benefits of markerless systems in terms of accessibility and user-friendliness, their validity and correspondence with reference systems, such as IMUs, remain under examination.

Despite growing interest in these technologies, there is a lack of studies that evaluate agreement between IMU and markerless systems and their ability to capture clinically meaningful biomechanical changes associated with pain and functional outcomes in patients with CNLBP. Additionally, the extent to which improvements in hip mobility reduce pain and disability remains uncertain. It is crucial that movement assessment in CNLBP evaluates symptom-provoking conditions, as pain is frequently triggered asymmetrically and may be associated with dysfunction in a specific lower limb during functional activities such as hip flexion. Despite this, most previous studies have not explicitly focused on the symptomatic limb during pain-provoking movement, thereby limiting the clinical interpretation of biomechanical findings.

This gap indicates the need for clinically grounded, technology-driven approaches that integrate objective biomechanical assessment with symptom-specific movement evaluation.

This study aimed to examine the association between changes in hip flexion and changes in low back pain in patients with chronic nonspecific low back pain (CNLBP), focusing specifically on the symptomatic lower limb in which lumbar pain is provoked during active hip flexion. A secondary aim was to evaluate the agreement between IMU- and markerless-derived hip flexion measurements under clinically relevant, pain-provocation conditions reflective of symptom-specific movement.

The present study combines repeated longitudinal biomechanical assessment with IMU and markerless motion capture analysis in order to investigate clinically relevant movement adaptations during rehabilitation in patients with CNLBP. In addition to examining associations between symptom-provoking hip flexion and pain-related outcomes, the study also evaluates the practical agreement and applicability of wearable and vision-based motion analysis systems under rehabilitation conditions.

The hypothesis was that improvements in hip flexion of the affected limb would be associated with reductions in low back pain intensity. Furthermore, it was hypothesized that IMU- and markerless-based measurements would present a strong correlation but modest agreement due to the intrinsic methodological distinctions between the two methods.

## 2. Materials and Methods

### 2.1. Study Design

A longitudinal repeated-measures approach was used to assess the association between changes in hip flexion and low back pain in patients with CNLBP. To identify substantial changes in both mobility and pain in patients with CNLBP, a maximum intervention period of 6 weeks was set. Prior rehabilitation studies have shown that structured exercise programs of comparable length are adequate to elicit considerable improvements in pain and function in this population [17,18]. However, to allow for individual variation in recovery, the intervention was designed with flexible termination criteria. More specifically, the program could be finished earlier if the participants did not undergo any symptom provocation, which was defined as a Visual Analog Scale (VAS) score of 0 for >2 consecutive sessions and no pain in the previously involved limb during movement execution. Data were systematically collected at each session during the intervention period to enable longitudinal monitoring of both biomechanical and clinical outcomes, with each participant completing 2–3 therapeutic sessions per week [19,20]. Ethical approval was granted by the Scientific Council (Bioethics and Ethics oversight) of Attikon University General Hospital, affiliated with the National and Kapodistrian University of Athens (Protocol No. 20, 23 January 2026).

### 2.2. Sample Size Calculation

An a priori power analysis was carried out using G*Power 3.1.9.7 (Heinrich-Heine-Universität Düsseldorf, Düsseldorf, Germany) for the primary correlation analysis of changes in symptomatic hip flexion range-of-motion and changes in pain intensity. A two-tailed Pearson correlation model was selected, with α = 0.05 and a statistical power of 0.80. Based on previous research with a similar biomechanical–clinical design [21] a large effect size (r = 0.50) was assumed. The analysis indicated that a minimum sample size of 29 participants was required [21].

### 2.3. Participants

The initial number of patients assessed for low back pain was 67 (37 women, 30 men). Inclusion criteria were non-specific lumbar spine pain, absence of pathological radiological findings on Magnetic Resonance Imaging (MRI), duration of low back pain exceeding 6 months, and no positive findings on lower-limb neurodynamic tests. Non-musculoskeletal pathological conditions, such as kidney disease presenting with low back pain and cancer, as well as lumbar pain attributable to anatomical or pathological alterations of the spine (e.g., scoliosis, spondylolisthesis, spondylosis, bone fractures, or neurological symptoms), were excluded. A total of 31 participants were excluded from the study: 3 due to spondylolysis, 9 due to sciatica, 2 due to radiological findings (scoliotic deformity), 1 due to spinal fracture, 13 due to lumbar spondyloarthropathy (Modic type II) [22], and 3 due to osteoporosis. The final sample consisted of 36 patients with CNLBP (22 women, 14 men). All participants were fully informed about the purpose and procedures of the study prior to participation. Written informed consent was obtained from all participants in the study, in line with ethical guidelines. The demographic characteristics of the participants are presented in Table 1.

### 2.4. Outcome Measures

To assess participants’ functional capacity, the Oswestry Disability Index (ODI) was used, one of the most extensively documented and widely used instruments for evaluating functional disability associated with low back pain [23]. In the present study, the validated Greek version of the assessment instrument was used. The ODI was administered during the first session of the therapeutic exercise intervention, prior to the initiation of the program, and during the final session, to evaluate changes in participants’ functional capacity in relation to the progression of the physiotherapeutic exercise program.

In parallel, pain intensity was assessed using the Visual Analog Scale (VAS), which was administered at each treatment session to allow continuous monitoring of patient progress. Participants were asked to rate the intensity of the pain they experienced in the lumbar region on a 10 cm straight line, where 0 represented “no pain” and 10 represented “maximum unbearable pain” [24]. The measurement was completed twice in each session: initially, prior to the therapeutic exercise, and immediately after its completion, to capture the intervention’s immediate influence on subjective pain feeling.

The Oswestry Disability Index (ODI) is scored from 0 to 100%, with higher scores indicating greater functional disability related to low back pain. In general, scores of 0–20% represent minimal disability, 21–40% moderate disability, 41–60% severe disability, and values above 60% indicate very severe functional limitation [9,25].

The Visual Analogue Scale (VAS) ranges from 0 to 10, where 0 indicates no pain and 10 represents the maximum imaginable pain intensity. Reductions in VAS scores are generally interpreted as clinically meaningful improvements in pain perception in patients with chronic musculoskeletal conditions [26].

ODI scores were collected at two time points only: prior to the initiation of the rehabilitation program and at the final treatment session following completion of the intervention period. In contrast, VAS scores were recorded during every treatment session, both immediately before and immediately after the therapeutic intervention.

Similarly, IMU and markerless hip flexion measurements were acquired repeatedly throughout the intervention period during symptom-provoking hip flexion assessments.

Pain onset was operationally defined as the point during active hip flexion at which participants first reported the onset of familiar lumbar pain or discomfort. Participants were instructed to slowly raise the symptomatic lower limb and verbally indicate the moment symptoms first appeared. At that point, the movement was terminated, and the corresponding hip flexion angle was recorded during the same movement trial using both the IMU and markerless motion capture systems.

Therefore, the study included both baseline-to-post-intervention comparisons and repeated longitudinal observations collected across treatment sessions.

All participants performed three repetitions of each therapeutic exercise prior to initiating the full intervention program to identify movements that provoked lumbar pain. All participants reported pain during single-leg hip flexion (knee-to-chest lower back stretch) assisted by the upper limbs, specifically in one of the two lower limbs. Consequently, the statistical analysis included 12 left-leg and 24 right-leg hip flexion range-of-motion (ROM) measurements, as these were the symptomatic extremities in which lumbar pain was elicited during hip flexion. The symptomatic limbs were captured using both IMU and markerless motion capture systems, and measurements were collected at the onset of pain during movement. This method ensured that the kinematic data accurately reflected clinically relevant conditions associated with pain provocation.

ODI and VAS values were used in the statistical analyses to examine their association with changes in hip flexion, both in terms of improved functional ability and reduced pain.

### 2.5. Intervention

Participants were positioned supine on a stable surface (a therapeutic exercise treatment table), with the upper limbs resting alongside the body and the lower limbs flexed at the knees. Participants wore form-fitting clothing in order to prevent errors in joint recognition by the mobile phone camera.

The therapeutic program consisted of seven exercises, all performed from a supine position with the knees flexed on a treatment table. The upper limbs were positioned alongside the body, with the palms resting on the mattress and the fingers in slight tension. All participants performed 3 familiarization repetitions. The exercises were as follows:

* Single-leg hip flexion (knee-to-chest lower back stretch) assisted by the upper limbs.

* Double-leg hip flexion (knee-to-chest lower back stretch) assisted by the upper limbs.

* Controlled alternating lateral rotations of the pelvis and lumbar spine with bent knees, without lifting the thoracic spine and with the shoulder girdle stabilized (Lower Trunk Rotations-LTR).

* Bridge/Shoulder Bridge from a supine position with both lower and upper limbs stabilized on the treatment table.

* Abdominal exercise with chest lift elevation assisted by the upper limbs up to the point where the scapulae begin to lift off the table, while the lower limbs remain in triple flexion.

* Supine Hip Abduction with Resistance Band (moderate-resistance elastic loop), with the lower limbs in triple flexion and stabilized on the table while holding the lumbar spine and pelvis in a neutral position.

* Isometric Hip Adduction with Pilates Ball/Adductor Squeezes, maintaining triple flexion of the lower limbs with the feet stabilized on the table while keeping the lumbar spine and pelvis in a neutral position.

For each exercise, 2 sets of 10 repetitions were performed with an inter-set rest period of 60 s.

After completing the exercise program, electrotherapy was applied, specifically Transcutaneous Electrical Nerve Stimulation (TENS) [27]. Four electrodes were positioned paraspinally at the lumbar spine levels (L1–L5), 2–3 cm from the vertebral column, in a BOX configuration, to effectively cover the region of localized pain. For most types of pain, electrodes are placed around the painful area so that the induced paresthesia can be directed toward the region of discomfort [28]. A conventional TENS mode was used, with a pulse width of 80–120 μs and a frequency of 80–100 Hz. The intensity was adjusted to the maximum painless sensory level, and the application duration was 15 min. Simultaneously with the TENS application, cryotherapy was also applied. The cold pack was covered with a protective sleeve to prevent direct contact with the patient’s skin and avoid possible cold burns. The main purpose was to facilitate muscle recovery after the program and achieve temporary analgesia, which is produced through neurophysiological mechanisms related to the Gate Control Theory of Pain [28]. The above physiotherapeutic intervention reflects a form of effective conservative usual care commonly applied in the management of chronic non-specific low back pain. This strategy was chosen to create a clinically relevant and standardized treatment framework rather than to evaluate the effectiveness of a specific therapeutic procedure. The main aim of this study was to examine the relationships between changes in hip mobility and their association with pain and functional impairment during symptom-inducing conditions, as previously described.

### 2.6. Motion Analysis

Although laboratory-based optical motion capture systems are considered the gold standard for biomechanical analysis, the present study focused on the comparison of two clinically applicable and scalable motion assessment approaches under rehabilitation conditions. IMUs were selected as the reference comparator because inertial sensor systems have demonstrated strong validity and reliability in previous rehabilitation and movement-analysis studies, while also offering practical advantages for routine clinical use outside laboratory environments [19,20,29,30].

**Figure 1:**
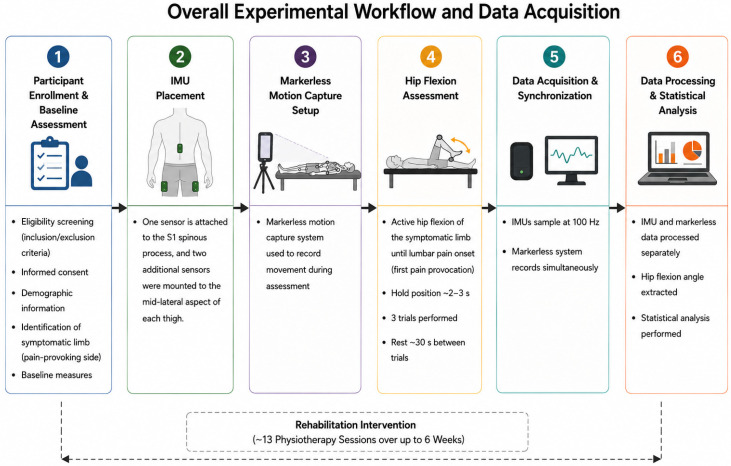
Overall experimental workflow and data acquisition process. The diagram illustrates participant enrollment and baseline assessment, IMU placement, markerless motion capture setup, hip flexion assessment, repeated longitudinal data acquisition during rehabilitation sessions, and subsequent data processing and statistical analysis.

To improve measurement consistency, all assessments were performed using a standardized acquisition protocol, identical participant positioning, fixed camera placement, and concurrent acquisition of IMU and markerless data during the same movement trials. All measurements were conducted by the same examiner under consistent environmental conditions throughout the intervention period. An overview of the experimental workflow and data acquisition process used in the present study is presented in Figure 1.

#### 2.6.1. Markerless System

The evaluation of active hip flexion range-of-motion constitutes a fundamental element in both clinical and research applications related to mobility, flexibility and their association with low back pain. Markerless motion capture systems enable the extraction of kinematic data without the need for physical markers [28,31,32]. This application utilizes computer vision and machine learning technologies to estimate joint angles based on data obtained from a mobile phone camera [32,33,34]. To enhance measurement accuracy and enable cross-validation of the method, Inertial Measurements Units (IMUs) were placed at specific anatomical locations on the lumbar region and lower limbs.

The markerless system adopted advanced pose estimation models based on TensorFlow Lite and the BlazePose architecture developed by Google. The system detects and records key points of the lower limbs (hip, knee and ankle joints) in real time from camera images. The mobile phone camera used by the application (iOS or Android, ≥1080p resolution, 60 fps) was placed on a stable tripod, at a distance of 2.5 m and a height of approximately 0.8–1.0 m from the floor, in the sagittal plane, with the optical axis parallel to the plane of motion.

The markerless motion capture system utilized a mobile phone camera (iPhone 15 Pro, Apple Inc., Cupertino, CA, USA) operating at 60 frames per second with a resolution of 1920 × 1080 pixels. The camera was positioned on a stable tripod at a distance of 2.5 m from the participant and at a height of approximately 0.8–1.0 m from the floor, with the optical axis aligned perpendicular to the sagittal plane of motion in order to minimize perspective distortion.

**Figure 2:**
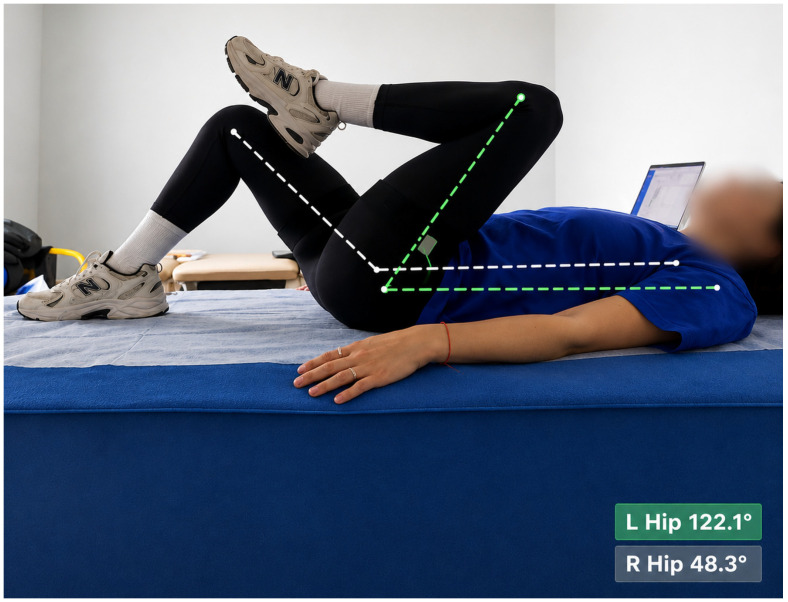
Experimental setup of the markerless motion capture system during active hip flexion assessment. The mobile phone camera was positioned in the sagittal plane to capture lower-limb motion during symptom-provoking hip flexion.

Hip flexion angles were estimated using the BlazePose pose estimation framework implemented through TensorFlow Lite. Joint positions were identified based on two-dimensional anatomical landmark tracking of the shoulder, hip, knee, and ankle joints (Figure 2). Hip flexion was calculated as the angle formed between the trunk and thigh segments in the sagittal plane at the point of symptom provocation.

A global laboratory coordinate orientation was maintained throughout all recordings, with participants positioned consistently relative to the camera. No additional smoothing filters were applied beyond the internal temporal stabilization incorporated within the pose estimation framework.

Potential sources of measurement variability included optical perspective distortion, inaccuracies in two-dimensional joint center estimation, partial landmark occlusion, soft tissue movement, lighting variability, and limitations related to depth estimation from monocular video acquisition [35,36].

Because the markerless system relied on monocular two-dimensional video acquisition, out-of-plane motion and depth-related estimation errors may have contributed to the observed systematic underestimation of hip flexion angles.

#### 2.6.2. Inertial Measurement Units (IMUs)

Three QSense, CE-approved for range-of-motion data capture, inertial measurement units (IMUs) (QSense Motion software, version 2.2.1, The Netherlands) were used to quantify hip flexion. Each sensor incorporated a tri-axial accelerometer, gyroscope, and magnetometer, enabling the capture of three-dimensional motion data. Data were recorded via Bluetooth connectivity, at a sampling frequency of 100 Hz (raw stream up to 400 Hz) and stored in CSV format for subsequent processing and analysis.

Sensor data fusion was performed using a nine-degrees-of-freedom (NDoF) algorithm that combined accelerometer, gyroscope, and magnetometer signals to estimate sensor orientation [37]. This process was additionally optimized by applying Kalman filtering techniques to eliminate noise and account for sensor drift [38,39]. Euler angles, which indicate joint motion in three-dimensional space, were used to express the final kinematic outputs.

A standardized calibration procedure was conducted before each data collection to ensure alignment between the sensor coordinate system and the participant’s anatomical reference frame. Magnetic field mapping was also implemented to minimize the effects of environmental magnetic interference, which could potentially reduce magnetometer accuracy and impair orientation estimation.

**Figure 3:**
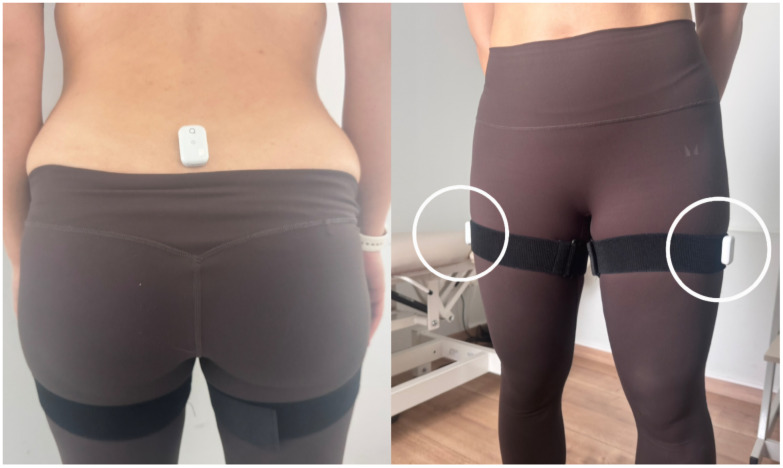
Placement of inertial measurement units (IMUs) during hip flexion assessment. One sensor was positioned over the S1 spinous process, while two additional sensors were placed on the mid-lateral aspect of each thigh.

The hip range-of-motion was captured by positioning the IMUs at predefined anatomical landmarks. To capture hip joint motion, one sensor was attached to the S1 spinous process, and two additional sensors were mounted on the mid-lateral aspect of each thigh. The S1 sensor was attached using hypoallergenic double-sided adhesive tape, and the thigh sensors were secured with elastic straps to assure stability during movement (Figure 3).

IMU data were recorded during active hip flexion trials, as described in Section 2.7. Two familiarization trials were completed prior to data collection to ensure correct movement execution. Subsequently, two repetitions of active hip flexion were recorded for each limb, with movement performed up to the point of pain onset in the lumbar region. The maximum angle reached at the moment of pain provocation was extracted for each repetition, and the mean value was used for analysis.

Data acquisition was performed at a sampling frequency of 100 Hz. The recorded signals were subsequently processed to extract the hip joint’s Euler angles. During these recording trials, concurrent acquisition measurements were obtained using both the IMU system and the markerless motion capture system, as described in the following section. With this concurrent data acquisition, the two measurement approaches could be directly compared, as hip flexion angles were recorded under identical movement conditions.

Quality control measures were implemented during data collection. After each recording session, the data were reviewed for potential irregularities or artifacts, including those caused by sensor misalignment or magnetic interference. Recalibration was performed as needed, and measurements were repeated to ensure data reliability [19,20].

### 2.7. Movement Execution Procedure

Standardized settings were used to test bilateral active hip flexion with knee flexion. Participants were instructed to perform slow, controlled hip flexion, raising the affected limb until the onset of lumbar pain, then returning to the initial position. The maximum hip flexion angle at the pain trigger location was held for five seconds. The contralateral asymptomatic limb was assessed under identical settings and functioned as a control. A 5 s rest time was provided between trials, and two repetitions were performed.

The markerless motion capture system automatically extracted the maximum angle between the trunk and the thigh in the sagittal plane at the moment of pain onset by recording it during each movement cycle. During the same movement trial, IMU-based data were also collected under identical conditions, allowing direct comparison between the two systems.

### 2.8. Statistical Analysis

A statistical analysis was performed to examine the relationship between clinical outcomes and variations in hip flexion. Descriptive statistics for hip flexion range-of-motion (mean ± standard deviation) were calculated at baseline and after the intervention. Furthermore, functional disability (ODI) and pain intensity (VAS) were documented and incorporated into the correlation analysis.

Pearson correlation coefficients were initially calculated as descriptive measures to explore associations between hip flexion measurements and clinical outcomes. Because repeated measurements were obtained across rehabilitation sessions, linear mixed-effects models were additionally applied as the primary inferential approach to account for within-subject dependency. Participant ID was entered as a random intercept to account for within-subject dependency, while changes in hip flexion were included as fixed effects in models examining associations with pain-related outcomes. Statistical significance was set at *p* < 0.05.

Agreement between hip flexion measurements obtained from the IMU and the markerless system was further evaluated using Bland–Altman analysis, including calculation of the mean difference (bias) and the limits of agreement (LoA).

Statistical analyses were performed using IBM SPSS Statistics 31 (IBM Corp., Armonk, NY, USA). For the linear mixed-effects analysis, restricted maximum likelihood (REML) estimation was applied, with participant-specific random intercepts included to account for repeated within-subject observations across rehabilitation sessions. Statistical significance was set at *p* < 0.05.

## 3. Results

A total of 36 participants (14 males and 22 females) contributed repeated longitudinal observations throughout the intervention period, with a mean of 13 physiotherapy sessions per participant. The mean age was 50.7 ± 5.7 years, and the mean body mass index (BMI) was 25.3 ± 2.7 kg/m^2^.

Participants completed a mean of 13 physiotherapy sessions (SD ± 3.45) until the predefined completion criteria were met.

### 3.1. Changes in Hip Range-of-Motion and Patient-Reported Outcomes

Hip joint flexion significantly increased (*p* = 0.00003), from 118.2° (SD = 2.79) at baseline to 130.1° (SD = 1.48) post-intervention, as measured by IMUs, and from 110.3° (SD = 2.55) to 120.5° (SD = 2.13) (*p* = 0.0002), as measured by the Markerless System.

Functional disability (ODI) decreased from 28.5 (SD = 3.03) at baseline to 12.4 (SD = 1.74) post-intervention; however, this change did not reach statistical significance (*p* = 0.095). Although the mean reduction numerically reflects an improvement from moderate toward mild disability, the wide variability in individual responses—as reflected in the non-significant *p*-value—indicates that this group-level change should be interpreted with caution rather than as definitive evidence of functional improvement.

### 3.2. Relationship Between Markerless System-Derived Hip Flexion and Pain Outcomes

**Figure 4:**
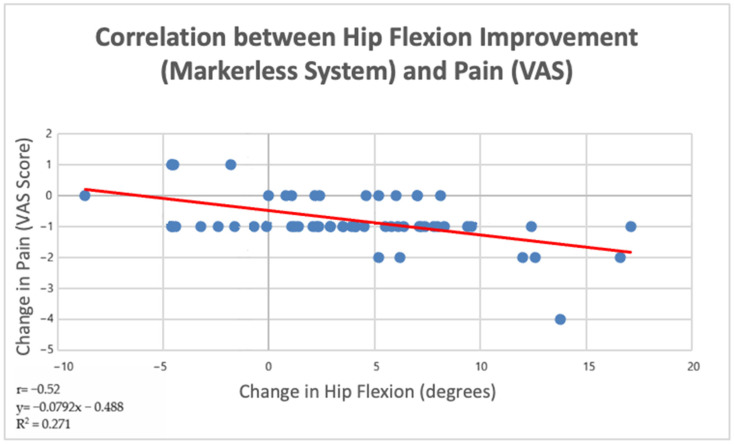
Correlation between changes in hip flexion (markerless system) and pain (VAS). These findings represent descriptive association patterns, whereas repeated-measures relationships were further examined using linear mixed-effects models.

A moderate negative correlation was found between changes in hip flexion measured by the markerless system and changes in pain (VAS) (r = −0.52, *p* = 0.001, 95% CI [−0.73, −0.23]). This indicates that greater improvements in hip flexion were associated with greater reductions in pain following the therapeutic exercise intervention (Figure 4).

Scatter plot illustrating the relationship between changes in hip flexion measured using the markerless system and changes in pain intensity (VAS). Each point represents a repeated observation obtained during the rehabilitation period. A moderate negative correlation was observed (r = −0.52), indicating that improvements in hip flexion were associated with reductions in pain.

### 3.3. Relationship Between IMUs-Derived Hip Flexion and Pain Outcomes

**Figure 5:**
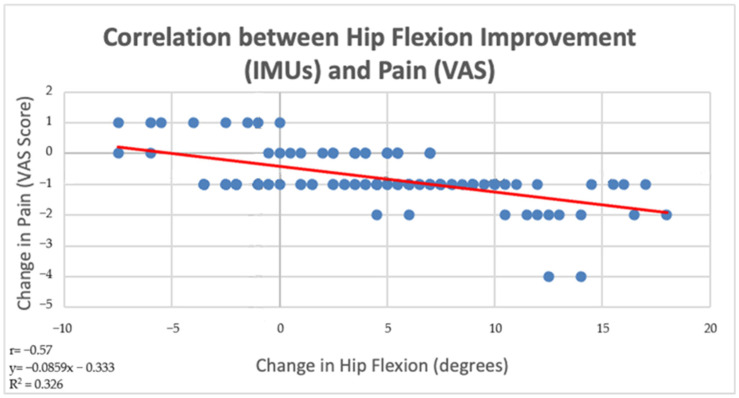
Correlation between changes in hip flexion (IMU) and pain (VAS). Scatter plot illustrating the relationship between changes in hip flexion measured using the IMU system and changes in pain intensity (VAS). Each point represents a repeated observation obtained during the rehabilitation period. A moderate-to-strong negative correlation was observed (r = −0.57), indicating that greater improvements in hip flexion were associated with greater reductions in pain. The Pearson correlations should be interpreted as exploratory associations, while mixed-effects analyses were used to account for repeated observations across sessions.

A moderate-to-strong negative correlation was observed between changes in hip flexion measured by the IMU and changes in pain (VAS) (r = −0.57, *p* < 0.001, 95% CI [−0.76, −0.30]). This indicates that greater improvements in hip flexion were associated with greater reductions in pain following the therapeutic exercise intervention (Figure 5).

### 3.4. Relationship Between Markerless-Derived Hip Flexion and Functional Disability (ODI Score)

**Figure 6:**
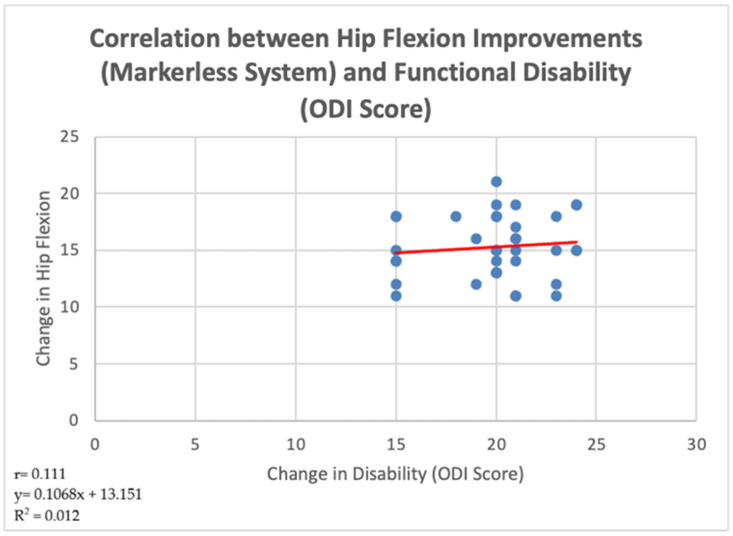
Correlation between changes in hip flexion (markerless system) and changes in disability (ODI score). A negligible positive correlation was found (r = 0.111, R^2^ = 0.012), suggesting no significant relationship between hip mobility changes and disability outcomes. To further address within-subject repeated measurements, additional linear mixed-effects analyses were performed.

A negligible and non-significant positive correlation was observed between changes in hip flexion (markerless system) and changes in disability (ODI score), as indicated by a very weak association (r = 0.111, *p* = 0.52, 95% CI [−0.23, 0.41], R^2^ = 0.012). The regression analysis (y = 0.1068x + 13.151) suggests that variations in hip flexion were not meaningfully related to changes in functional disability. Overall, these findings indicate that improvements in hip mobility, as measured by the markerless system, did not correspond to significant changes in ODI scores within this sample (Figure 6).

### 3.5. Relationship Between IMU-Derived Hip Flexion and Functional Disability (ODI Score)

**Figure 7:**
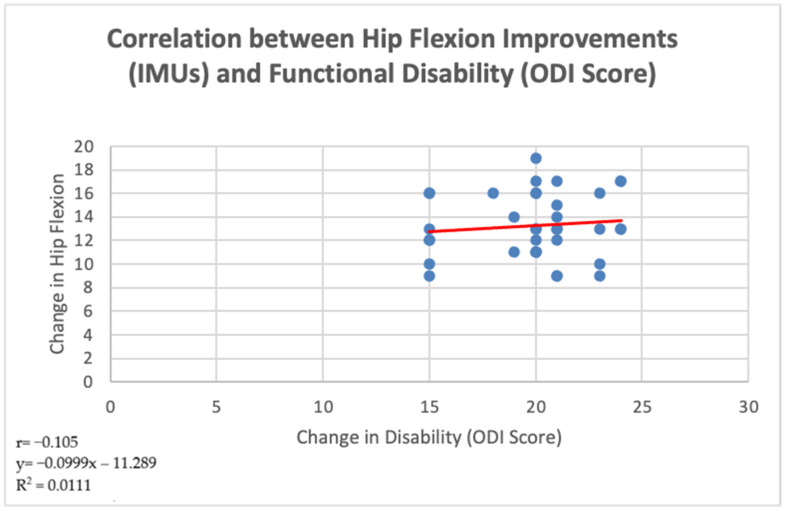
Correlation between changes in hip flexion (IMU-derived measurements) and changes in disability (ODI score). A negligible negative correlation was observed (r = −0.105, R^2^ = 0.011), suggesting no meaningful association between improvements in hip mobility and functional disability. Although correlation analyses were initially performed descriptively, repeated-measures inference was additionally evaluated using mixed-effects modeling approaches.

A negligible and non-significant correlation was observed between changes in hip flexion (IMU-derived) and changes in disability (ODI score), as indicated by a very weak negative association (r = −0.105, *p* = 0.54, 95% CI [−0.40, 0.24], R^2^ = 0.011). The regression analysis (y = −0.0999x − 11.289) suggests that improvements in hip flexion were not meaningfully associated with changes in functional disability. Overall, these findings indicate that, within this sample, hip mobility improvements did not translate into measurable changes in ODI scores (Figure 7).

The observed reduction in ODI scores suggests an improvement from moderate functional disability at baseline toward mild disability levels following the intervention period.

### 3.6. Sensitivity Analysis Between Markerless- and IMU-Derived Changes in Hip Flexion and Changes in Pain Intensity

**Figure 8:**
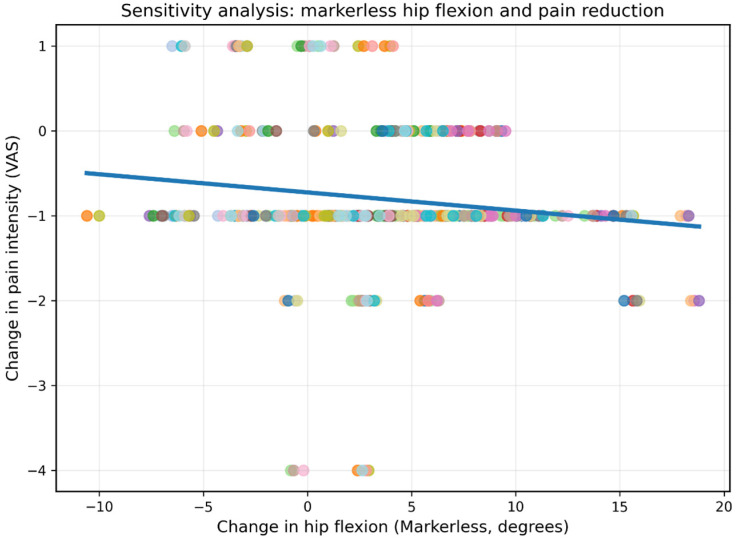
Sensitivity analysis demonstrating the association between markerless-derived changes in hip flexion and changes in pain intensity (VAS) across repeated rehabilitation sessions. Each color represents repeated observations from an individual participant. The solid regression line represents the linear mixed-effects model fit, accounting for within-subject repeated measurements. Improvements in hip flexion were significantly associated with reductions in pain intensity, although with a smaller effect size compared to IMU-derived measurements.

**Figure 9:**
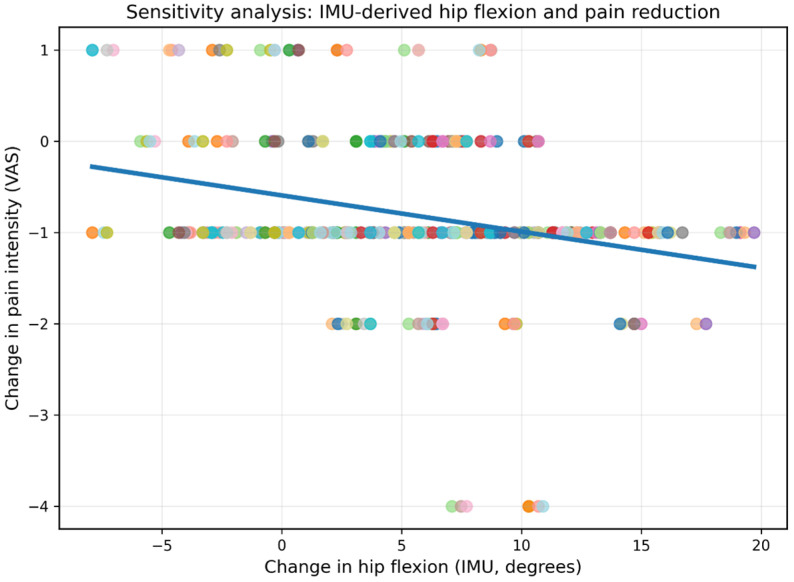
Sensitivity analysis demonstrating the association between IMU-derived changes in hip flexion and changes in pain intensity (VAS) across repeated rehabilitation sessions. Each color represents repeated observations from an individual participant. The solid regression line represents the linear mixed-effects model fit, accounting for within-subject repeated measurements. Greater improvements in hip flexion were associated with greater reductions in pain intensity.

Linear mixed-effects analysis demonstrated that improvements in hip flexion remained significantly associated with reductions in pain intensity after accounting for repeated within-subject observations across treatment sessions. Specifically, IMU-derived hip flexion improvements were significantly associated with pain reduction (β = −0.046, 95% CI [−0.063, −0.029], *p* < 0.001). Similarly, markerless-derived hip flexion improvements also demonstrated a significant association with pain reduction (β = −0.022, 95% CI [−0.038, −0.006], *p* = 0.007), although with a smaller effect size compared to IMU measurements (Figure 8 and Figure 9).

### 3.7. Agreement Between Μarkerless- and IMU-Derived Hip Flexion Measurements

The markerless system demonstrated a strong positive correlation with the IMU measurements (Pearson correlation coefficient analysis: r = 0.87, *p* < 0.001, 95% CI [0.75, 0.93]), indicating that both systems captured similar kinematic patterns of hip flexion across sessions.

**Figure 10:**
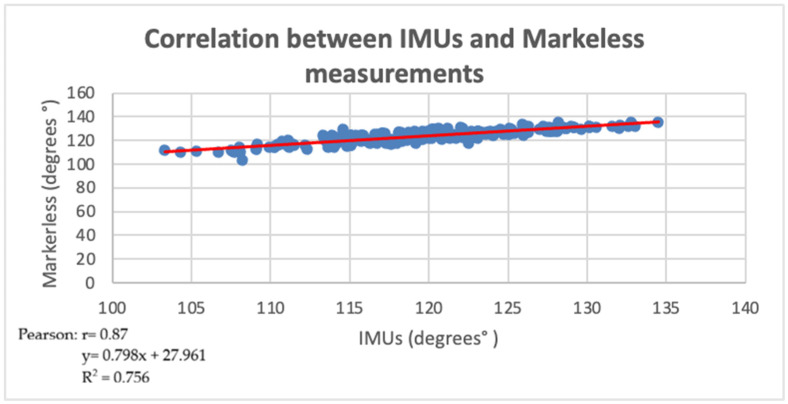
Correlation between IMU and markerless measurements of hip flexion. Scatter plot illustrating the relationship between the angles measured using an inertial measurement unit (IMU) and a markerless motion capture system. Each point represents a single measurement. A strong positive correlation was observed (r = 0.87).

Scatter plot showing the relationship between IMU and markerless measurements (r = 0.87). The solid line represents the linear regression fit (Figure 10).

**Figure 11:**
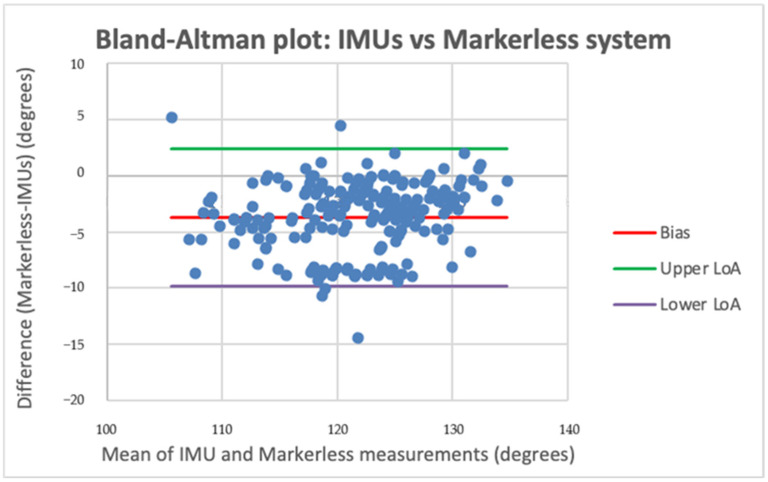
Bland–Altman analysis of agreement between IMU and markerless measurements. Bland–Altman plot showing agreement between IMU measurements and those from the markerless system. The solid red line represents the mean difference (bias = −3.67°), while the green and blue lines indicate the limits of agreement (2.43° to −9.79°). Most observations lie within the limits of agreement, indicating moderate agreement between the two methods.

However, the Bland–Altman analysis revealed a mean difference (bias) of −3.67°, indicating that the markerless system systematically underestimated the right and left hip angles relative to the IMU. The limits of agreement ranged from −9.79° to 2.43°, indicating moderate agreement between the two measurement methods and suggesting that, although the markerless approach can reliably track changes in movement, it cannot be considered interchangeable with IMU measurements. Most data points were distributed within the limits of agreement, although a small number of outliers were observed. The dispersion pattern suggests a tendency for greater variability at lower measurement values, without clear evidence of proportional bias (Figure 11).

## 4. Discussion

The present findings should be interpreted within a symptom-specific, pain-provocation movement framework, as all measurements were obtained from the limb that provoked lumbar pain during hip flexion [40].

Due to the observational longitudinal design and the lack of a control group, randomization, and blinding, these findings should be interpreted strictly as associations. While improvements in hip flexion were associated with reductions in pain, causality cannot be established. Other factors, such as natural symptom fluctuation, therapeutic exposure, patient expectations, neuromuscular adaptation, or psychosocial influences, may have contributed to the observed changes.

Importantly, the mixed-effects analyses demonstrated findings consistent with the original correlation analyses, supporting the robustness of the observed associations between symptom-provoking hip flexion and pain reduction across statistical approaches.

These findings are interpreted as associations rather than causal relationships. This approach enhances the clinical relevance of the results by reflecting the functional conditions under which pain is generated, rather than relying on asymptomatic or averaged bilateral measurements.

The current results indicate that hip flexion was markedly enhanced after the therapeutic exercise program, thus facilitating the restoration of hip mobility in individuals with CNLBP [39]. This improvement is clinically significant, as limited hip mobility has been consistently linked to altered lumbopelvic mechanics, increased lumbar spine stress, and the persistence of low back pain [6,41,42,43,44].

Enhancements in hip flexion were marginally linked with reductions in pain, as indicated by negative correlations for both the markerless system (r = −0.52) and IMU-derived data (r = −0.57). These results are in line with previous studies that have found that improved hip mobility reduces pain by improving movement efficiency and reducing compensatory movement in the lumbar spine [5,42,43]. Moreover, similar relationships between biomechanical gains and pain relief have been reported in musculoskeletal rehabilitation contexts [18,45,46].

Nonetheless, despite substantial improvements in hip range-of-motion, no significant association was detected between improvements in hip flexion and changes in functional impairment (ODI), as indicated by minimal correlations in both IMU-derived (r = −0.105) and markerless assessments (r = 0.111). The evident disconnect between biomechanical enhancement and reported functional recovery is clearly documented and underscores the multifaceted nature of chronic non-specific low back pain (CNLBP) [17,45]. Functional disability is not affected by physical impairments but also by psychosocial and behavioral factors, which may explain the poor correlation observed in the current study [3,46,47,48].

The identification of an association between increased hip flexion and pain supports the idea that movement impairments contributing to chronic non-specific low back pain may be limb-specific and should be assessed in the presence of pain-provoking conditions, which may indicate a localized biomechanical adaptation in the symptomatic limb.

This study offers an in-depth analysis of the validity and clinical utility of markerless and IMU-based motion capture systems. The solid correlation between the two systems (r = 0.87) suggests that markerless methodologies can accurately capture movement patterns, consistent with other validation studies [49,50,51]. Bland–Altman analysis indicated modest agreement, with the markerless approach repeatedly underestimating hip flexion, underscoring that correlation does not imply interchangeability.

The interpretation that the observed bias primarily reflects markerless underestimation rather than IMU overestimation was based on the extensive validation literature supporting IMU-derived kinematic measurements, which generally demonstrate relatively small angular errors and strong agreement with reference motion capture systems. In contrast, markerless systems estimate joint angles indirectly through image-based anatomical landmark prediction and are inherently more sensitive to projection distortions, depth estimation limitations, and joint-center localization inaccuracies [52,53,54].

Nevertheless, because the present study did not include an external gold-standard optical motion capture reference system, the observed bias should be interpreted as a relative systematic disagreement between the two measurement approaches rather than definitive evidence of absolute markerless underestimation.

The systematic underestimation observed in the markerless measurements may be explained by several geometric and algorithmic limitations inherent to monocular computer vision systems. Because joint angles were estimated from two-dimensional image projections, inaccuracies related to depth perception and out-of-plane motion may have influenced landmark localization. In addition, pose estimation algorithms such as BlazePose estimate joint centers indirectly through probabilistic anatomical landmark prediction rather than direct biomechanical tracking, which may introduce cumulative angular estimation errors.

Further sources of variability may include optical perspective distortion, partial landmark occlusion, soft tissue movement, and temporal smoothing processes incorporated within pose estimation algorithms. These factors may collectively contribute to systematic angle underestimation, particularly at larger hip flexion angles or during movements involving subtle trunk compensation strategies [14,30,35,36,53].

Importantly, many of these limitations are theoretically correctable through improved camera calibration procedures, multi-camera three-dimensional reconstruction methods, enhanced biomechanical constraints, and next-generation AI-based pose estimation frameworks [14,35,53].

The slight disparity between the two methods indicates that markerless motion capture can nevertheless yield clinically significant data, especially in contexts where user-friendliness, cost-effectiveness, and short setup durations are essential. Recent research has highlighted the promise of markerless systems as feasible instruments for remote monitoring and digital rehabilitation [13,55,56].

Significantly, the consistency of findings across both measurement systems strengthens the validity of these observations, indicating that the associations with pain reduction and the lack of association with disability are not artifacts of measurement error but reflect underlying clinical mechanisms.

Overall, these results highlight that while hip mobility restoration is a key biomechanical outcome, it should not be considered in isolation when evaluating rehabilitation success. Pain is a multifactorial construct influenced by biomechanical, neuromuscular, and psychosocial factors [45]; therefore, a comprehensive assessment combining objective kinematic data with patient-reported outcomes is essential.

### Limitations

Several limitations should be considered when interpreting the findings of the present study.

Because of the observational design, potential confounding factors may have influenced the observed associations between hip mobility and pain reduction. Variables such as baseline symptom severity, psychosocial influences, individual rehabilitation adherence, neuromuscular adaptation, and treatment responsiveness were not independently controlled for within the statistical models. Therefore, the findings should be interpreted as longitudinal associations rather than independent causal relationships [57].

Formal test–retest reliability analysis was not performed because the primary aim of the study was to examine longitudinal biomechanical associations and agreement between clinically applicable motion analysis systems rather than to establish device reliability.

IMUs provide accurate kinematic data; however, due to soft tissue artifacts (STAs) arising from the relative motion of the skin and underlying skeletal structures, their attachment to the skin may result in measurement errors [19,20,58,59,60]. Previous studies have shown that these errors are generally small, typically below 5% of the full range-of-motion, and are further minimized during controlled, low-intensity movements such as those performed in this study [61,62,63]. In addition, IMU-based measurements have demonstrated strong agreement with reference systems, supporting their validity in assessing spinal and hip kinematics.

Markerless motion capture systems introduce another layer of unpredictability, as they use computer vision techniques to estimate joint positions. Measurement accuracy can be affected by factors such as camera placement, lighting conditions, and occlusions, while shortcomings in depth perception and joint center estimate can lead to systematic underestimation of joint angles [49,50,51]. These restrictions likely contributed to the modest agreement between the markerless and IMU systems. In contrast, IMU-based systems provide direct kinematic data and have been shown to be highly sensitive to changes in joint angles [11,12], which may explain the slightly stronger correlation between IMU-derived hip flexion and reduced pain.

Chronic non-specific low back pain is a heterogeneous condition involving multiple biomechanical, neuromuscular, and psychosocial mechanisms [4]. In the present study, participants were analyzed as a single clinical CNLBP cohort without subgroup stratification based on movement–control impairment classifications or pain mechanism subtypes. Therefore, potential biomechanical differences between distinct pathological or motor-control profiles may not have been fully captured. Future studies should incorporate classification-based subgrouping approaches in order to better characterize subtype-specific movement adaptations and improve the interpretability of biomechanical associations.

Because the markerless system relied on monocular two-dimensional image acquisition, depth-related estimation limitations and projection distortions may have contributed to the observed systematic bias [35,36,53]. Future developments using multi-camera systems and three-dimensional pose reconstruction may improve angular accuracy and reduce estimation variability.

The absence of a laboratory-based optical motion capture reference system limits the ability to determine absolute measurement accuracy. Future studies incorporating simultaneous comparison with gold-standard three-dimensional motion capture systems are warranted.

Lastly, the study was conducted in a controlled setting with a small sample size and no control group, which limits the extent to which the results can be generalized. To improve measurement accuracy and practical application, future studies should examine the combined use of IMUs and markerless technologies across larger populations.

## 5. Conclusions

In conclusion, this study’s findings indicate that improvements in hip mobility correlate with reductions in low back pain under specific symptomatic conditions. Although IMUs deliver superior measurement accuracy, markerless systems offer a pragmatic, scalable option for detecting clinically significant changes. These findings support the adoption of objective motion analysis into rehabilitation practices and underscore the promise of developing technology for accessible, data-driven musculoskeletal evaluation.

Future research needs to focus on advancing algorithmic robustness, enhancing calibration procedures, and exploring hybrid sensor-fusion approaches to optimize accuracy and agreement across measurement modalities.

## Figures and Tables

**Table 1:** Baseline demographic and anthropometric characteristics of the study participants, including total sample size, gender distribution, age, and body mass index (BMI) (mean ± standard deviation).

Variable	Category	Value (n or Mean ± SD)
Total Number		36
Gender	Male	14
	Female	22
Age		50.7 ± 5.7
BMI		25.3 ± 2.7

## Data Availability

The data presented in this study are available on request from the corresponding author due to privacy restrictions.
